# In Vivo Imaging of Thyroid Cancer with ^99m^Tc-TR1401 and ^99m^Tc-TR1402: A Comparison Study in Dogs

**DOI:** 10.3390/jcm10091878

**Published:** 2021-04-26

**Authors:** Filippo Galli, Michela Varani, Chiara Lauri, Giuseppe Campagna, Lajos Balogh, Bruce D. Weintraub, Mariusz W. Szkudlinski, Armando Bartolazzi, Isabella Manni, Giulia Piaggio, Alberto Signore

**Affiliations:** 1 Nuclear Medicine Unit, Department of Medical-Surgical Sciences and of Translational Medicine, Faculty of Medicine and Psychology, “Sapienza” University of Rome, 00161 Rome, Italy; varanimichela@gmail.com (M.V.); chiara.lauri@uniroma1.it (C.L.); gius.campagna@gmail.com (G.C.); alberto.signore@uniroma1.it (A.S.); 2 National “Frederic Joliot Curie” Research Institute for Radiobiology and Radiohygiene, 1221 Budapest, Hungary; balogh.lajos@osski.hu; 3 Trophogen Inc., Rockville, MD 20850, USA; bweintraub@trophogen.com (B.D.W.); mszkudlinski@trophogen.com (M.W.S.); 4 Pathology Research Laboratory, St. Andrea University Hospital, 00189 Rome, Italy; a.bartolazzi@lilt.it; 5 Department of Research, Diagnosis and Innovative Technologies, IRCCS Regina Elena National Cancer Institute, 00144 Rome, Italy; isabella.manni@ifo.gov.it (I.M.); giulia.piaggio@ifo.gov.it (G.P.)

**Keywords:** rhTSH, TR1402, TR1402, thyroid cancer, molecular imaging

## Abstract

Differentiated thyroid cancer (DTC) cells may lose NIS expression and iodine uptake, but usually express TSH receptors (TSHR). Therefore, the aim of our study was to compare two radiolabeled superagonist TSH analogues for DTC imaging. These analogues (namely TR1401 and TR1402) have a higher TSHR binding affinity than recombinant human TSH (Thyrogen ^®^). Radiolabeling was performed with technetium-99m using an indirect method via HYNIC conjugation and was followed by in vitro quality controls and binding assay on TSHR-positive cell lines (ML-1). An in vitro binding assay was also performed and compared with radiolabeled human recombinant TSH. In vivo imaging was performed in four dogs with spontaneous follicular thyroid carcinoma with solid poorly differentiated areas with ^99m^Tc-TR1401 SPECT/CT, ^99m^Tc-TR1402 SPECT/CT, and [^18^F]FDG PET/CT on different days within 2 weeks. TR1401 and TR1402 were labeled with high specific activity (8.3 ± 1.2 MBq/µg) and retention of their biological activity and structural integrity. Both agonists were able to efficiently bind TSHR receptors expressed by cell lines with dissociation constants (Kd) of 2.7 nM for ^99m^Tc-TR1401 and 0.5 nM for ^99m^Tc-TR1402 compared with ^99m^Tc-Thyrogen (Kd = 8.4 nM). In tumor-targeting experiments, a focal uptake was observed in dogs with spontaneous intraglandular thyroid carcinoma, in which TSHR expression was confirmed by immunohistochemistry. ^99m^Tc-TR1402 provided higher T/B than ^99m^Tc-TR1401 and [^18^F]FDG (12.9 ± 1.3, 10.2 ± 0.7, and 3.8 ± 0.6, respectively; all *p* < 0.001). Given these results, ^99m^Tc-TR1402 appears to be a useful tool for in vivo imaging of thyroid cancer.

## 1. Introduction

The incidence of thyroid cancer has been increasing over the years because of both the improvement in diagnostic techniques and increases in risk factors [1,2]. For this reason, more and more variants of thyroid cancer are diagnosed with several degrees of differentiation [3]. Differentiated thyroid cancer (DTC) generally has a good prognosis; the therapeutic approach includes total thyroidectomy followed by radioiodine ablation of the remnant tissue. This is due to the presence of the sodium/iodide symporter (NIS), which accumulates the radioisotope in both normal and malignant cells [4]. Therefore, iodine-131 uptake is considered a good prognostic marker, having been used as a first-line treatment for local or metastatic DTC for years [5,6]. Thyroid cancer patients generally have a favorable prognosis, but some may show distant metastases at the time of first diagnosis (1–4%) or during follow-up (7–23%) [7,8]. In addition, thyroid cancer cells gradually lose their NIS expression [9] and become radioiodine refractory DTC (RR-DTC) [10]. These malignancies require alternative diagnostic and therapeutic approaches and show low or no iodine uptake (either ^124^I or ^131^I) but high thyroglobulin (Tg) values; staging is actually performed by PET with [^18^F]FDG [11]. Thus, it is possible to detect residual disease and plan the most appropriate therapy (thermoablation, radiotherapy, chemotherapy, or tyrosine kinase inhibitors) [12]. In these cancers, it was demonstrated that the TSHR, although not functional, can still be expressed on the plasma membrane [13]. This concept led some groups to exploit this feature for therapeutic or diagnostic approaches [14,15].

Our group demonstrated the possibility to target the TSHR with a specific radiopharmaceutical based on a new superagonist recombinant human (rh)TSH analogue [16]. One of the main advantages of this approach is the possibility of performing a preliminary scan before the thyroidectomy to identify possible local or distant metastases that lost the capacity to uptake radioiodine, but are still positive to the TSHR. Given the previous promising results with ^99m^Tc-TR1401 and the availability of a new superagonist rhTSH analogue (TR1402), we aimed to compare these two molecules radiolabeled with technetium-99m in dogs with spontaneous intraglandular thyroid cancer to select the best candidate to be translated in humans. [^18^F]FDG PET/CT was also performed in all dogs.

## 2. Materials and Methods

### 2.1. Labeling of Superagonist rhTSH Analogue TR1402 with Technetium-99m

The superagonist rhTSH analogue TR1402 was produced at Trophogen, Inc. (Rockville, MD, USA) by site-directed mutagenesis techniques in stably transfected Chinese hamster ovary (CHO) cells and purified by a combination of dye, ion exchange, and gel-filtration HPLC. The TR1402 molecule contains four arginine substitutions in the α subunit of the glycoprotein in comparison with the wild-type molecule [17,18]. Indirect labelling of a highly purified TR1402 molecule was performed by conjugation with the bifunctional chelator succinimidyl-6-hydrazinonicotinate hydrochloride (HYNIC). The conjugation and radiolabeling with technetium-99m of the TR1402 analogue were performed as previously described for TR1401 [16]. 

### 2.2. In Vitro Quality Controls

Quality controls were performed using both instant thin-layer chromatography-silica gel (ITLC-SG) strips (Pall Life Sciences, Port Washington, NY, USA) and thin-layer chromatography silica gel (TLC-SG) plates (Pall Life Sciences, Port Washington, NY, USA). Strips and plates were read with a linear radio-scanner (Bioscan Inc., Poway, CA, USA) to calculate the LE of ^99m^Tc-HYNIC-TR1402. Mobile phases were 0.9% NaCl (Rf: ^99m^Tc-O_4_^−^ 0.9; ^99m^Tc-TR1402 0.1; colloids 0.1) and a NH_3_:H_2_O:EtOH (1:5:3) solution (Rf: ^99m^Tc-O_4_^−^ 0.9; ^99m^Tc-TR1402 0.9; colloids 0.1). Radiochemical purity was also determined after size exclusion chromatography. Stability assays were performed by diluting ^99m^Tc-HYNIC-TR1402 (100 μL) in fresh human blood serum (900 μL) or 0.9% NaCl solution (900 μL). Vials were incubated at 37 °C and the radiochemical purity was measured at 1, 3, 6, and 24 h by ITLC analysis. In addition, a cysteine challenge assay was performed by incubating the radiolabeled TR1402 at 37 °C for 60 min with increasing concentrations of cysteine ranging from a 1000:1 (cysteine:TR1402) to 0.1:1 molar ratio. For each time point, radiochemical purity was evaluated by ITLC as described above.

### 2.3. Cell Culture and In Vitro Binding Studies

Human ML-1 cells were cultured as previously described [16]. Briefly, cells were grown in DMEM plus 10% FCS, 100 mM sodium pyruvate, 2 mM l-glutamine, 1 mg/mL glucose, 3.7 g/L NaHCO_3_, and antibiotics. Measurements of cell uptake and retention of radiolabeled TR1402 were performed in vitro using LigandTracerTM (Ridgeview Instruments AB, Uppsala, Sweden), a semi-automatic device [19]. ML-1 cells (10^6^) were seeded in a defined area of a tilted Petri dish and incubated in a humified incubator at 37 °C and 5% CO_2_ for 24 h. The dish was then placed in the LigandTracer and rotated for 15 min to remove weakly attached cells. After one gentle wash, 2 mL of 30 nM radiolabeled TR1402 was added to the cell culture medium and the dish started to rotate for 1 h. When maximum uptake was reached, the radiolabeled solution was replaced with culture medium without labeled TR1402 to evaluate the release of radioactivity from cells. Data were analyzed with GraphPad Prism (GraphPad Software Inc, San Diego, CA, USA) and a binding/release curve was drawn to calculate kon, koff, and Kd values. The same experiment was carried out using radiolabeled TR1401 and Thyrogen to compare the affinity of the radiopharmaceuticals for the TSHR [16].

### 2.4. Studies in Dogs with Spontaneous Thyroid Carcinoma 

Four dogs with a spontaneous, palpable cervical mass were selected for the study and underwent routine [^18^F]FDG PET/CT (scan at 1 h post injection of approximately 250 MBq) after fasting for 12 h. Scans were performed on a PET/CT scanner with 7 min 3-dimensional static acquisitions per bed position. Three beds were acquired for each dog. The total scan time was 21 min. Blood tests and fine needle aspiration biopsies were performed to assess the thyroidal origin of the lesion (thyroid carcinoma). Then, after informed consent from their owner, each animal was studied by SPECT/CT imaging with both ^99m^Tc-HYNIC-TR1401 and ^99m^Tc-HYNIC-TR1402 under deep anesthesia (scans at 3 h post injection of 220–250 MBq, 24–30 µg). Scanning conditions were 20 s/frame, 60 frames, 1 frame every 6°, with 20% energy window centered at 140 keV. We selected 3 h as the imaging time point based on results previously obtained [16]. For target and background calculation, irregular regions of interest (ROIs) were drawn over the tumor, over the contra-lateral thyroid lobe (guided by CT transaxial image), and over the vertebral body, of the same transaxial section, as the background. All dogs underwent surgery to excise the lesion for histology and immunohistochemistry (IHC) using a rabbit anti-dog TSHR (LifeSpan Biosciences, Seattle, WA, USA). T/B ratios are shown as mean ± standard deviation (SD). The Shapiro–Wilk test was used to verify the normality of distribution of residuals. Homoscedasticity was verified by Levene and Brown–Forsythe tests. Comparison between FDG, TR1401, and TR1402 was performed following a general linear mixed model (GLIMMIX) procedure, considering Gaussian function as distribution and identity as link. Post hoc analysis was performed by Tukey’s method. A *p*-value < 0.05 was considered statistically detectable. All statistical analyses were performed by using SAS v.9.4 (SAS Institute Inc., Cary, NC, USA). Animal studies were approved by the local ethics committee (approval no. PE/EA/208-1/2016).

## 3. Results

### 3.1. Labelling of TR1402 with Technetium-99m and Quality Controls 

Highest labeling efficiency was obtained when the analogue was conjugated with an 8:1 starting HYNIC:TR1402 ratio. MSR results demonstrated that 2.1, 4.6, and 6.1 molecules of SHNH were bound per molecule of analogue when using a 4:1, 8:1, and 12:1 HYNIC:TR1402 ratio, respectively. Each other experiment was performed using the 8:1 ratio; the best results were obtained by radiolabeling the TR1402 (40 µg) with 370 MBq of pertechnetate, 200 μL of tricine (1.1 mM), and 5 μL of SnCl2 (50 nM). These conditions produced an LE of 96 ± 2% and <5% of colloids after 10 min of incubation. After SEC purification, radiochemical purity was >99% and specific activity was 8.3 ± 1.2 MBq/µg. Radiolabeled TR1402 was stable for up to 24 h in human serum, a 0.9% NaCl solution at 37 °C, and in cysteine solutions. 

### 3.2. In Vitro Binding Studies

**Figure 1:**
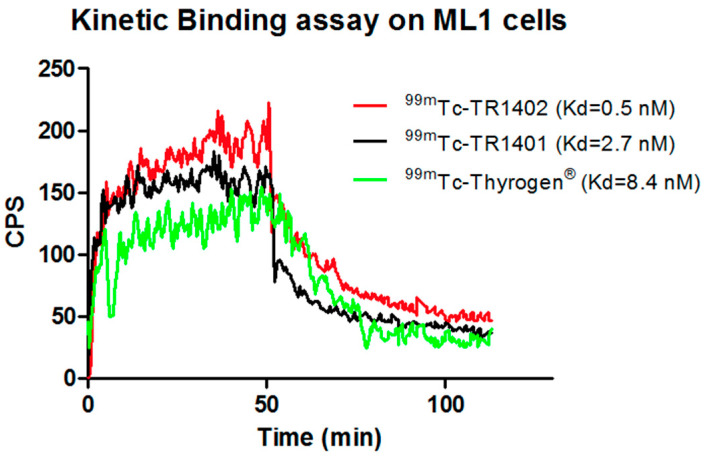
Real-time kinetic binding assay on ML1 cells with ^99m^Tc-TR1402 (red line), ^99m^Tc-TR1401 (black line), and ^99m^Tc-Thyrogen (green line). Highest affinity was detected for ^99m^Tc-TR1402.

ML-1 cells showed fast radiopharmaceutical uptake, per LigandTracer™, reaching a plateau within 20 min and a slow dissociation from TSHR with time. The calculated Kd for ^99m^Tc-TR1401, ^99m^Tc-TR1402, and ^99m^Tc-Thyrogen was 2.7, 0.5, and 8.4, respectively (Figure 1). 

### 3.3. Case Study in Dogs with Spontaneous Thyroid Carcinoma 

**Figure 2:**
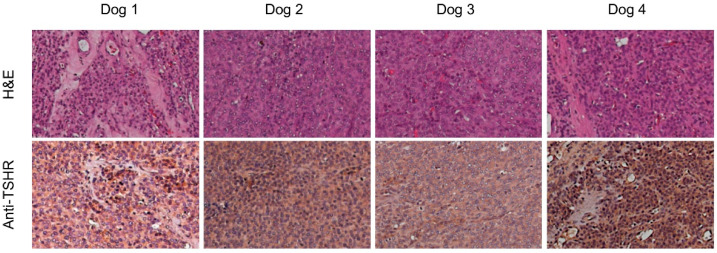
Histological section of excised lesion from the 4 dogs stained with hematoxylin/eosin (**top** panels), revealing a solid/trabecular follicular thyroid carcinoma (200× magnification). Histological section stained with an anti-dog-TSHR antibody reveals homogeneous staining in almost all cancer cells (**bottom** panels).

Three dogs were clinically hyperthyroid and one dog was euthyroid. Routine hematological and biochemical parameters were all within the normal range. The results of post-surgical histology showed a follicular thyroid carcinoma with trabecular/solid less-differentiated areas and pT2 Nx Mx in all dogs (Figure 2). Tumors were highly positive at immunohistochemistry for TSHR (Figure 2).

**Figure 3:**
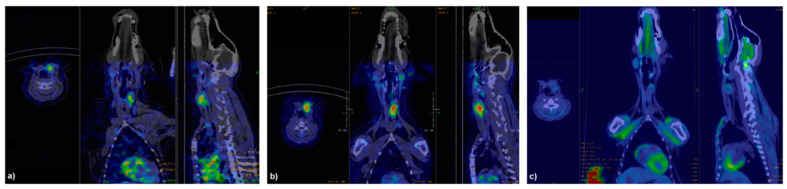
^99m^Tc-TR1401 (**a**), ^99m^Tc-TR1402 (**b**), and [^18^F]FDG (**c**) PET-CT images in dog 1 with a spontaneous trabecular/solid follicular thyroid cancer. Dog was hyperthyroid and showed high tumor uptake of ^99m^Tc-TR1401 and ^99m^Tc-TR1402. This dog showed the lowest FDG uptake in the tumor.

**Figure 4:**
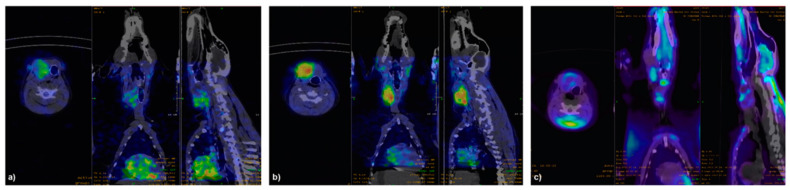
^99m^Tc-TR1401 (**a**), ^99m^Tc-TR1402 (**b**), and [^18^F]FDG (**c**) PET-CT images in dog 4 with a spontaneous trabecular/solid follicular thyroid cancer. Images clearly show high tumor uptake of ^99m^Tc-TR1401 and ^99m^Tc-TR1402 (similar to the dog in Figure 3). This dog showed the highest FDG uptake in the tumor.

In all dogs, SPECT/CT images with labeled agonists showed high uptake in the thyroid lobe corresponding to the thyroid nodule, with no significant uptake in the contralateral lobe (Figure 3 and Figure 4).

**Figure 5:**
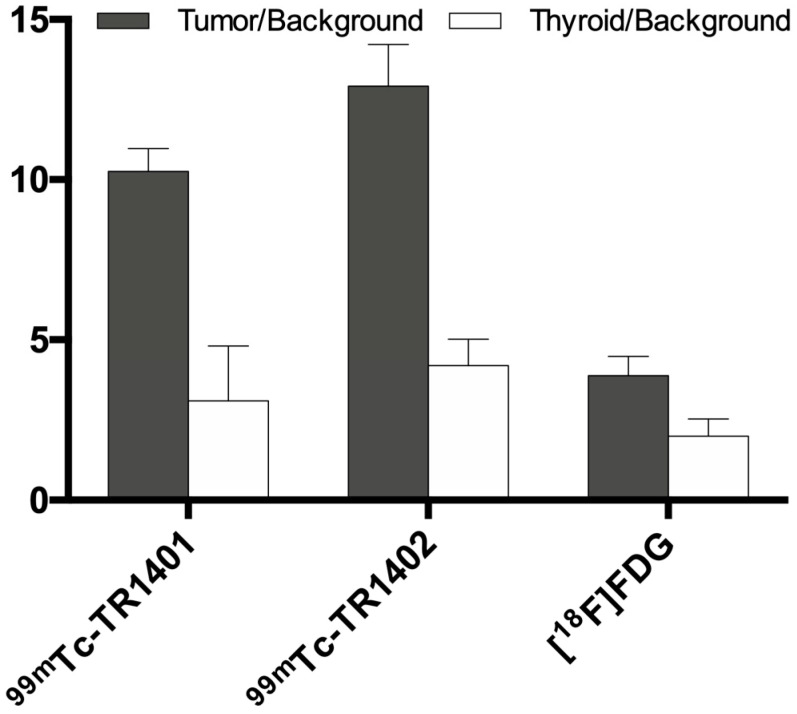
Tumor/background ratio (grey bars, mean ± SD) and thyroid/background ratio (white bars, mean ± SD) of ^99m^Tc-TR1401, ^99m^Tc-TR1402, and [^18^F]-FDG in studied dogs. For tumor/background ratio, the target was chosen on the thyroid nodule in the most representative axial sections; the background, of the same size, has been selected over the vertebral body of the same section. Thyroid/background ratio was calculated drawing a ROI on the contralateral lobe. Both ^99m^Tc-TR1401 and ^99m^Tc-TR1402 significantly accumulated in the tumor more than FDG (*p* < 0.0001), and ^99m^Tc-TR1402 significantly accumulated more than ^99m^Tc-TR1401 (*p* < 0.0001). The same applied when comparing tumor/background vs. thyroid/background ratios (all *p* < 0.0001). No significant differences were found when comparing thyroid/background ratios of different radiopharmaceuticals.

No focal uptake was found either in the regional lymph nodes or in other tissues, but in the liver and kidneys as a consequence of radiopharmaceutical metabolism. ^99m^Tc-TR1402 always showed more intense uptake than ^99m^Tc-TR1401. [^18^F]FDG uptake in tumors was of variable degree and distribution, not always matching with intratumoral distribution of labelled superagonists (Figure 3 and Figure 4). Radiolabeled TR1402 showed the highest T/B ratio in tumors compared with TR1401 or [^18^F]FDG (12.9 ± 1.3, 10.2 ± 0.7, and 3.8 ± 0.6, respectively; all *p* < 0.001), but not significantly different T/B in the contralateral thyroid lobe (4.2 ± 0.8, 3.1 ± 1.7, and 1.9 ± 0.5, respectively) (Figure 5).

## 4. Discussion

An accurate in vivo characterization of thyroid and extra-thyroid lesions remains a clinical issue. This is particularly important in patients in whom the loss of the NIS is often linked to the presence of distant metastases at the time of diagnosis [20]. These lesions can remain undetected when the first line therapy is performed, so patients develop more undifferentiated and malignant forms that will be hard to treat with conventional approaches. For this reason, the possibility of non-invasively detecting such lesions would help to define the most accurate first line therapeutic approach and follow-up with patients in a more accurate manner [21]. New target and diagnostic strategies are, therefore, being investigated [22]. In addition to targeting specific thyroid cancer cells, other markers like galectine-3 or TSHR can, possibly, be investigated as potential targets for cancer imaging [23,24]. In particular, from several studies, it emerged that the TSHR is expressed in human thyroid cancers with different degrees of differentiation [12,25], which is confirmed by our IHC results on excised tumors from dogs affected by thyroid cancer. We already showed the possibility of imaging thyroid cancer using iodine-radiolabeled Thyrogen [26], but the study was limited by its relatively low affinity for TSHR [27]. For this purpose, we developed new radiolabeled superagonist rhTSH analogues to test them in both murine models and dogs [16]. In this study, we radiolabeled TR1401 and TR1402, two superagonist rhTSH analogues, with technetium-99m using a well-established indirect approach and compared in vitro receptor binding affinity and in vivo uptake by thyroid tumors in dogs. 

Our previous results with ^99m^Tc-TR1401 showed the potential clinical use of this superagonist for imaging thyroid cancer cells [16]. In particular, given the availability of dogs with spontaneous thyroid cancer, we exploited the superiority of this model over mice with tumor xenografts. Murine models of cancer xenograft are somehow artificial and the lack of an appropriate tumor microenvironment does not always allow us to easily translate results into humans [28]. Dogs often develop spontaneous thyroid cancers with histological features very similar to human follicular cancer, and expressing TSHR. Several authors have investigated thyroid cancer in dogs by nuclear medicine procedures, but scans were limited to SPECT/CT with ^123^I, ^131^I, or ^99m^TcO_4_^−^ [29,30]. No data are available about FDG uptake in dogs with thyroid cancer or information about galectin-3 expression in this model for the lack of species-specific galectin-3 mAbs. Our previous results opened the door for the potential use of radiolabeled TSH superagonists for a pre-operative staging in patients with thyroid cancer and for post-operative follow-up. Before testing ^99m^Tc-TR1401 in humans, the availability of a new superagonist, TR1402, motivated us to radiolabel it and compare its behavior in vitro and in vivo in dogs compared with ^99m^Tc-TR1401 and [^18^F]FDG. In our study, dogs had a single intraglandular palpable mass and showed low [^18^F]FDG uptake and high ^99m^Tc-TR1401 and ^99m^Tc-TR1402 uptake in the tumor, with the latter showing the highest T/B ratio. In vitro, ^99m^Tc-TR1402 showed higher Kd than ^99m^Tc-TR1401 and ^99m^Tc-Thyrogen, confirming the higher affinity of the TR1402 for the TSHR expressed on human cell lines of thyroid cancer and explaining the high T/B ratios observed in vivo in dogs. The high specific activity and Kd of ^99m^Tc-TR1402 allowed us to inject a very low amount of protein (approximately 30 µg), resulting in a high target-to-background ratio with no biological effects; that, however, remains to be evaluated. Theoretically, the stimulation of the TSHR on metastatic lesions may lead to increased proliferation rate, tumor growth, and hormone release, as well as hyperthyroidism. For this reason, both the specific activity and affinity of TSH-based radiopharmaceuticals should be as high as possible. Finally, as shown by other groups, the TSHR can also be used as a therapeutic target [31], and we will explore the possibility to radiolabel the TR1402 analogue with beta-emitting isotopes, such as lutetium-177 or yttrium-90, for possible therapeutic applications. The possible limitations of this study are the low number of animals used (due to difficulty in recruiting volunteers). Furthermore, we did not investigate the possible presence of distant metastases since total body images were not acquired. Our study focused on the in situ thyroid tumor and contralateral thyroid lobe.

## 5. Conclusions

This study demonstrated that the radiolabeled superagonist rhTSH analogue (TR1402) has high affinity for the TSHR and, in dogs with spontaneous thyroid cancer, shows a high T/B ratio able to image an intrathyroidal cancer lesion. ^99m^Tc-TR1402 is a good candidate radiopharmaceutical to be translated in humans to evaluate its contribution as a noninvasive diagnostic tool for pre-operative staging of patients affected by thyroid cancer and their follow-ups. Moreover, in a veterinary setting, it could be an innovative technique to image dogs affected by thyroid cancer.

## Data Availability

The data presented in this study are available within this article. Any other data is available on request from the corresponding author.
